# Bilateral and Multiple Central Serous Chorioretinopathy Following COVID-19 Infection: A Case Report and Literature Review

**DOI:** 10.7759/cureus.23246

**Published:** 2022-03-17

**Authors:** W Mohd Mohd-Alif, Adnan Nur-Athirah, Maya Sapira Hanapi, Tengku Norina Tuan Jaffar, Ismail Shatriah

**Affiliations:** 1 Ophthalmology, Hospital Raja Perempuan Zainab II, Kota Bharu, MYS; 2 Ophthalmology and Visual Sciences, School of Medical Sciences, Universiti Sains Malaysia, Kubang Kerian, MYS; 3 Ophthalmology Clinic, Hospital USM, Universiti Sains Malaysia, Kubang Kerian, MYS; 4 Ophthalmology, Hospital Raja Perempuan Zainab Il, Kota Bharu, MYS

**Keywords:** psychological stress, corticosteroid treatment, central serous chorioretinopathy, central visual loss, covid-19 infection

## Abstract

Central serous chorioretinopathy (CSCR) following coronavirus disease 2019 (COVID-19) infection is rare. We describe an adult patient who survived a COVID-19 infection and received intravenous and oral corticosteroid treatment for three weeks. He presented three weeks post COVID-19 infection with central visual loss in both eyes for six days. Fundus examination showed multiple localized serous retinal detachments in both eyes. Optical coherence tomography (OCT) of the macula confirmed the presence of multiple areas of serous retinal detachment and pigment epithelial detachment. The patient was treated with topical non-steroidal anti-inflammatory eye drops and regained full visual recovery after three months. Corticosteroid treatment for COVID-19 and psychological stress induced by the disease are potential risk factors for the development of CSCR. Physicians should be aware of this side effect, as an early referral to an ophthalmologist for treatment is essential.

## Introduction

Coronavirus disease 2019 (COVID-19) is currently a global pandemic caused by a novel severe acute respiratory syndrome coronavirus 2 (SARS-CoV-2). Various ocular manifestations have been reported worldwide related to this disease, ranging from the eyelid, ocular surface, anterior segment, posterior segment, and neuro-ophthalmic involvement [1,2] and these may be the first signs of COVID-19 infection or develop weeks after recovery.

Central serous chorioretinopathy (CSCR) is characterized by serous retinal detachment with or without retinal pigment epithelium detachment. CSCR is common in young and middle-aged males, with a peak incidence at 45 years of age [3]. However, the association of CSCR with COVID-19 infection has not been widely reported. We present a unique case of post-COVID-19 infection presenting with bilateral and multiple CSCR.

## Case presentation

A 38-year-old man with no comorbidity was diagnosed with COVID-19 infection after a positive polymerase chain reaction swab test. He was admitted to a local hospital on day six of the illness due to worsening lethargy, fever, and cough. He received intravenous ceftriaxone 1 g daily and dexamethasone 4 mg three times daily over his nine-day stay at the hospital. The corticosteroid was changed to oral dexamethasone upon discharge, with a slow tapering dose for over two weeks.

The patient presented to the Ophthalmology Clinic 10 days after hospitalization with subacute onset of central visual loss in both eyes for six days. He described the symptom as multiple blackish spots increasing in size over time, affecting his central vision. There was no retro-orbital eye pain, redness, any history of recent vaccination, or ocular trauma. He was a non-smoker and denied having a similar symptom before.

**Figure 1:**
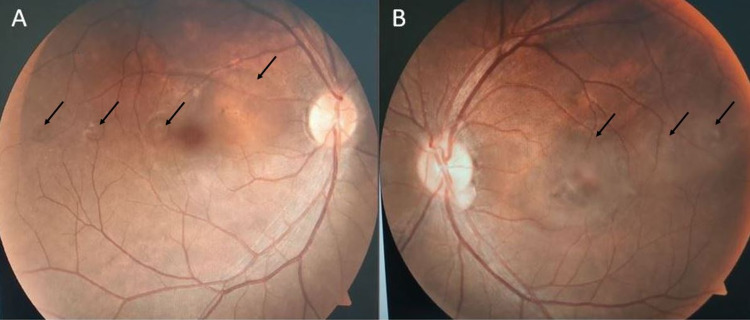
Fundus photograph demonstrating multiple localized areas of bilateral serous retinal detachment (black arrows) in right (A) and left (B) eyes

**Figure 2:**
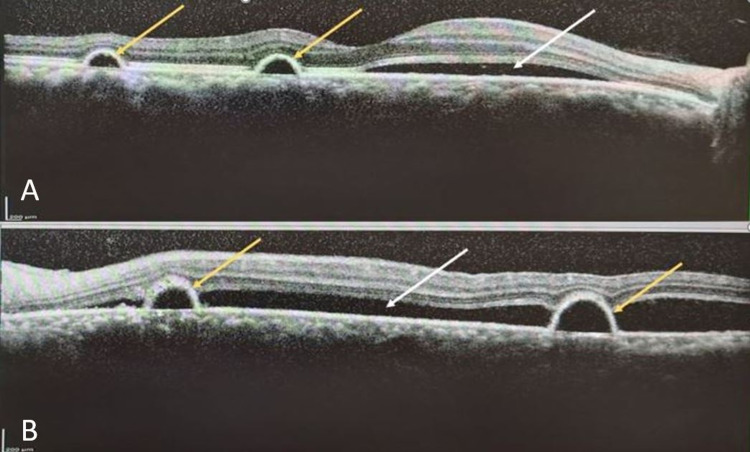
OCT scan across macula of right (A) and left (B) eyes illustrating serous retinal detachments (white arrow) with retinal pigment epithelial detachments (yellow arrows). Visual acuity was 6/6 (20/20 OD) and 6/12 (20/40 OS) Abbreviations: OCT, optical coherence tomography; OD, right eye; OS, left eye.

Examination showed that his visual acuity was 6/6 (20/20 OD) and 6/12 (20/40 OS), with no relative afferent pupillary defect. The anterior segment examinations were normal in both eyes. Fundus examination revealed multiple localized areas of serous retinal detachment of the macula in both eyes (Figure 1). Spectral-domain optical coherence tomography (OCT) of the macula confirmed the findings of serous retinal detachment with multiple areas of pigment epithelial detachment bilaterally (Figure 2).

**Figure 3:**
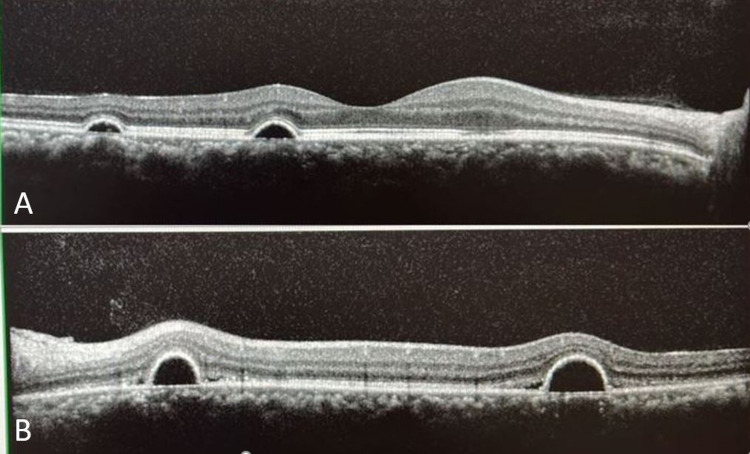
OCT scan across macula of right (A) and left (B) eyes showing improvement after one month. Visual acuity was 6/6 (20/20 OD) and 6/9 (20/30 OS) Abbreviations: OCT, optical coherence tomography; OD, right eye; OS, left eye.

**Figure 4:**
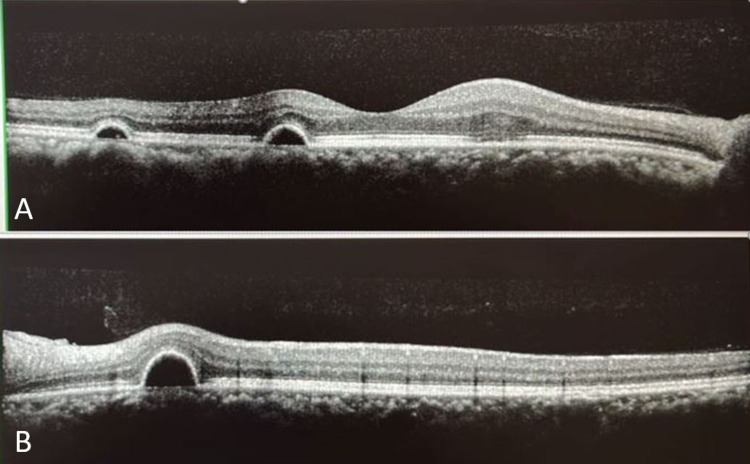
OCT scan across macula of right (A) and left (B) eyes showing persistence of pigment epithelial detachments after three months. Visual acuity was 6/6 (20/20 OD) and 6/6 (20/20 OS) Abbreviations: OCT, optical coherence tomography; OD, right eye; OS, left eye.

The oral corticosteroid was slowly tapered off to avoid possible sequelae of COVID-19 infection. The patient was prescribed topical nepafenac 0.1% three times daily for both eyes and advised for lifestyle modification and stress management. During follow-up after one month, he showed noticeable improvement in visual acuity and macular lesions (Figure 3). He regained full visual recovery three months after the initial presentation. However, the macula lesions did not achieve a complete resolution (Figure 4) and he was advised follow-up for further monitoring.

## Discussion

CSCR is one of the most common retinopathies worldwide [4]. However, the link between CSCR and the current COVID-19 pandemic has not been widely reported. There have been three published cases to date, all of which occurred unilaterally [5-7]. We present a unique case following COVID-19 infection involving bilateral and multiple CSCR. Table 1 summarizes the published reports on CSCR following COVID-19 infection [5-7], including our patient.

**Table 1:** Summary of published case reports of CSCR linked to COVID-19 Abbreviations: CSCR, central serous chorioretinopathy; OD, right eye; OS, left eye; OU, bilateral eyes; CF, counting fingers.

Authors/year	Age/gender	Laterality	COVID-19 infection timeline	Corticosteroid treatment	Presenting visual acuity	Treatment	Duration of visual recovery	Outcome
Sanjay et al./2021 [5]	42/female	OD	12 days post COVID-19	Oral dexamethasone and oral methylprednisolone	6/12	Nil	1 month	6/7.5
Amulya et al./2021 [6]	30/male	OD	2 weeks post COVID-19	Nil	CF	Nil	2 months	6/6
Goyal et al./2021 [7]	27/female	OS	2 weeks post COVID-19	Oral corticosteroid	6/18	Nil	Few weeks	6/6
Our patient/2022	38/male	OU	3 weeks post COVID-19	Oral and intravenous dexamethasone	OD 6/6, OS 6/12	Topical nepafenac	3 months	OD 6/6, OS 6/6

The case reported by Sanjay et al. was a 42-year-old female who developed multifocal CSCR in her right eye [5]. Amulya et al. described a 30-year-old male who developed CSCR in his right eye [6], while Goyal et al. reported a case of a 27-year-old female with CSCR in her left eye [7]. All three cases developed unilateral CSCR in about two weeks following COVID-19 infection [5-7] and received corticosteroid treatment, except for the case described by Amulya et al. [6]. Our patient developed multiple CSCR in both eyes three weeks after COVID-19 infection.

The exact pathogenesis of CSCR is still unclear; however, multiple risk factors have been linked to this disease including age, race, treatment with corticosteroids, emotional stress, type-A behavior, *Helicobacter pylori* infection, sleep apnea, pregnancy, organ transplantation, and autoimmune diseases [8]. In our case, the patient had two possible risk factors: psychological stress induced by COVID-19 infection and corticosteroid treatment.

The COVID-19 pandemic has caused a significant psychological impact on daily life. This includes increased rates of depression, anxiety, and stress [9]. We postulate that this is the major precipitating factor for CSCR in our patient. The pandemic affects the physical and mental health of COVID-19 patients silently.

Second, corticosteroids are known to counter the inflammation that can lead to lung injury which is why they are one of the main classes of medications used in the management of COVID-19. It is a proven fact that they reduce mortality and shorten the duration of hospitalization in patients infected with COVID-19 [10]. Our patient had been treated with a systemic corticosteroid for a total of three weeks. This may have played a causative or potentiating role in the occurrence of CSCR in this case.

The standard first-line treatment for CSCR is careful monitoring along with risk factor reduction. It has an excellent prognosis and follows a self-resolving natural course with the recovery of visual function. None of the above patients described in Table 1 received treatment for CSCR [5-7], except our patient. We started topical nepafenac in our patient in view of the multiple lesions and involvement of bilateral eyes. He showed full visual recovery with almost complete resolution of CSCR after three months, which is a slightly longer duration compared to other reported cases of CSCR following COVID-19 infection [5-7].

## Conclusions

CSCR can occur in patients following COVID-19 infection. Psychological stress and corticosteroid administration in the treatment of COVID-19 are the potential major risk factors for developing CSCR. Physicians managing patients with COVID-19 should be aware of this side effect, as an early referral for ophthalmology assessment is crucial.
